# Supplementary material for: Impacts on air dose rates after the Fukushima accident over the North Pacific from 19 March 2011 to 2 September 2015
Source: PLoS One. 2022 Aug 24;17(8):e0272937. doi: 10.1371/journal.pone.0272937 (PMC9401177; doi:10.1371/journal.pone.0272937)
Supplement: S1 Appendix — (PDF) [file pone.0272937.s001.pdf]

Impacts on air dose rates after the Fukushima accident over the North Pacific from 19 March 2011 to 2 September 2015: Supporting information S1 Appendix

Kuo-Ying Wang
Department of Atmospheric Sciences, National Central University, Chung-Li, Taiwan

Philippe Nedelec
Laboratoire d'Aérologie, Centre National de la Recherche Scientifique, Observatoire Midi-Pyrénées, 14 Avenue E. Belin, 31400 Toulouse, France

Hannah Clark
IAGOS-AISBL, 98 Rue du Trone, Brussels, Belgium

Neil Harris
Centre for Environment and Agricultural Informatics, Cranfield University, Cranfield, UK

Mizuo Kajino
Meteorological Research Institute (MRI), Japan Meteorological Agency (JMA), Tsukuba, Ibaraki, Japan

Yasuhito Igarashi
Division of Nuclear Engineering Science. Institute for Integrated Radiation and Nuclear Science, Kyoto University (KURNS), 2-chome, Asashiro-Nishi, Kumatori-cho, Sennan-gun, Osaka, 590-0494, Japan

Short title: AIR DOSE RATES OVER THE NORTH PACIFIC

Abstract. In this Supporting Information, we show additional figures and tables in support of the main text.

Table 1.

Table S1 shows list of published measurements of the air dose rates. **Table 2.**

Table S2 shows list simulations and measurements on the Tokyo Port calling ships (in units of μSv/h). **Figure 1.**

Figure S1 shows calibration of fourteen sensors for measurements of air dose rate. Each panel comprises five subpanels. The upper left shows the first comparison between the designated dose rates (Des Span) versus the measured dose rates (Obs Span). The open squares/crosses indicate that the observed values are higher/lower than the designated values. The upper right shows the calibration factors with respect to the designated dose rates. The open squares/crosses indicate that the calibration factors (the ratios of the designated to the observed spans) are greater/lower than one. The lower two sub-panels are the results from the second calibrations. The lowest subpanel shows a linear model of the calibration factors with respect to day. **Figure 2.**

Figure S2. shows time-series measurements of the air dose rates (in the unites of μSv/h) at the Tokyo Shinjuku[51] site (35.7065°N, 139.6979°E). (a) For the period from 12 to 31 March 2011. (b) For the period from 11 March to 31 December 2011. Horizontal red line indicates background level of 0.04 μSv/h. **Figure 3.**

Figure S3 shows measurements of the air dose rates (in the unites of μSv/h) and the model simulations of the ^{137}Cs and ^{131}I (in the units of (mBq/m^3, and converted μSv/h, respectively) at the Tokyo Shinjuku[51] site (35.7065°N, 139.6979°E) for the period from 11 to 31 March 2011. **Figure 4.**

Figure S4 shows comparison of the simulated and observed air dose rates on 14 March 2011. Left panels present large-scale, middle panels show magnified , and righ panels show Tokyo Port area views. The upper panels show results at 15 UT, central panels show resluts at 18UT, and the bottom panels show results at 21UT. The measurements at the corresponding times and locations are colored according to the air dose rates and encircled. **Figure 5.**

Figure S5 shows comparison of the air dose rates measured on the Tokyo Port calling ships (light blue color) and on the Pacific sailing ships (brown color) that had returned to the Tokyo Port.

Figure 6.

Figure S6 shows measurements of the air dose rates over the North Pacific from March 2011 to February 2012. The monthly data are arranged from the top left to righ to the right bottom.

Figure 7.

Figure S7 shows measurements of the air dose rates over the North Pacific from March 2012 to February 2013. The monthly data are arranged from the top left to righ to the right bottom.

Figure 8.

Figure S8 shows measurements of the air dose rates over the North Pacific from March 2013 to February 2014. The monthly data are arranged from the top left to righ to the right bottom.

Figure 9.

Figure S9 shows measurements of the air dose rates over the North Pacific from March 2014 to February 2015. The monthly data are arranged from the top left to righ to the right bottom.

Figure 10.

Figure S10 shows measurements of the air dose rates over the North Pacific from March to June 2015. The monthly data are arranged from the top left to righ to the right bottom.

Figure 11.

Figure S11 shows scatter plot analysis of the FDNPS radiocesium emission fluxes (black crosses) and measured Tsukuba radiocesium deposition fluxes (blue circles) versus the monthly mean air dose rates (pmean) on sailing ships. (a) North Pacific Ocean. (b) Northwest Pacific Ocean. (c) Northeast Pacific Ocean. (d) Northwest Atlantic Ocean.

Figure 12.

Figure S12 shows simulated dispersion of the radionuclides ^{137}Cs and ^{131}I (in the converted units of μSv/h) at 18 UT on each day from 11 to 30 March 2011. Days are ordered from left to right and from top to bottom).

Figure 13.

Figure S13 shows the spatial distribution of the scaled abosrbing aerosol index (AAI) from the OMI satellite. The results are presented on a horizontal resolution of 1

degree longitude-latitude grid system. The values on each grid represent the summation of the monthly AAI, from February 2011 to December 2015, and scaled by the AAI values on a grid covering the Tokyo port area.

Figure 14.

Figure S14 the same as in Figure S14, but for the Japan region, between 137°E and 146°E, and between 33°N and 42°N.

Figure 15.

Figure S15 shows air dose rates monitored at Hawaii (in the converted units of μSv/h) from 2017 to 2020. The average air dose rates are 0.032 μSv/h.

Figure 16.

Figure S16 shows the gamma gross count rates per minute monitored at Hawaii from 2010 to 2020. Monitored results from (a) channel range 02; (b) channel range 03; (c) channel range 04; (d) channel range 05; (e) channel range 06; (f) channel range 07; (g) channel range 08; (h) channel range 09. The vertical red line indicates the time of the FDNPS accident.

Figure 17.

Figure S17 shows the scaled gamma gross count rates per minute at Hawaii observed from 2010 to 2020. Results are scaled by the 2011 average values. (a) channel range 02; (b) channel range 03; (c) channel range 04; (d) channel range 05; (e) channel range 06; (f) channel range 07; (g) channel range 08; (h) channel range 09. The vertical red line indicates the time of the FDNPS accident.

Figure 18.

Figure S18 shows time-series measurements of the air dose rates (in dark brown color) on the sailing ships in a region containing Hawaii (between 170°W and 140°W, and between longitudes 10°N and 30°N; see also Figure 3). The air dose rates measured on the calling ships of the Tokyo Port are shown in sky blue color for comparison. The lowest horizontal green line shows the 2017-2020 average air dose rates of 0.034 μSv/h measured in Hawaii. After correcting for the latitudinal (spatial) effects of 1.4, the second horizontal green line (from the bottom) shows the estimated air dose rates of 0.048 μSv/h during 2011-2015 ($0.034 * 1.4 = 0.048$) in Hawaii. After correcting for the time (temporal) effects of 1/0.6, the third horizontal green line (from the bottom) shows the estimated air dose rates of 0.057 μSv/h during 2011-2015 in Hawaii

(0.034*1/0.60=0.057) in Hawaii. After correcting for both the time and the latitudinal effects, the top horizontal green lines shows the estimated air dose rates of 0.080 μSv/h during 2011-2015 (0.034 * 1.4 *1/0.60= 0.048) in Hawaii. We note that 0.080 μSv/h are close to the mean of the all PGGM measurements of 0.082 μSv/h over the north Pacific.

Figures

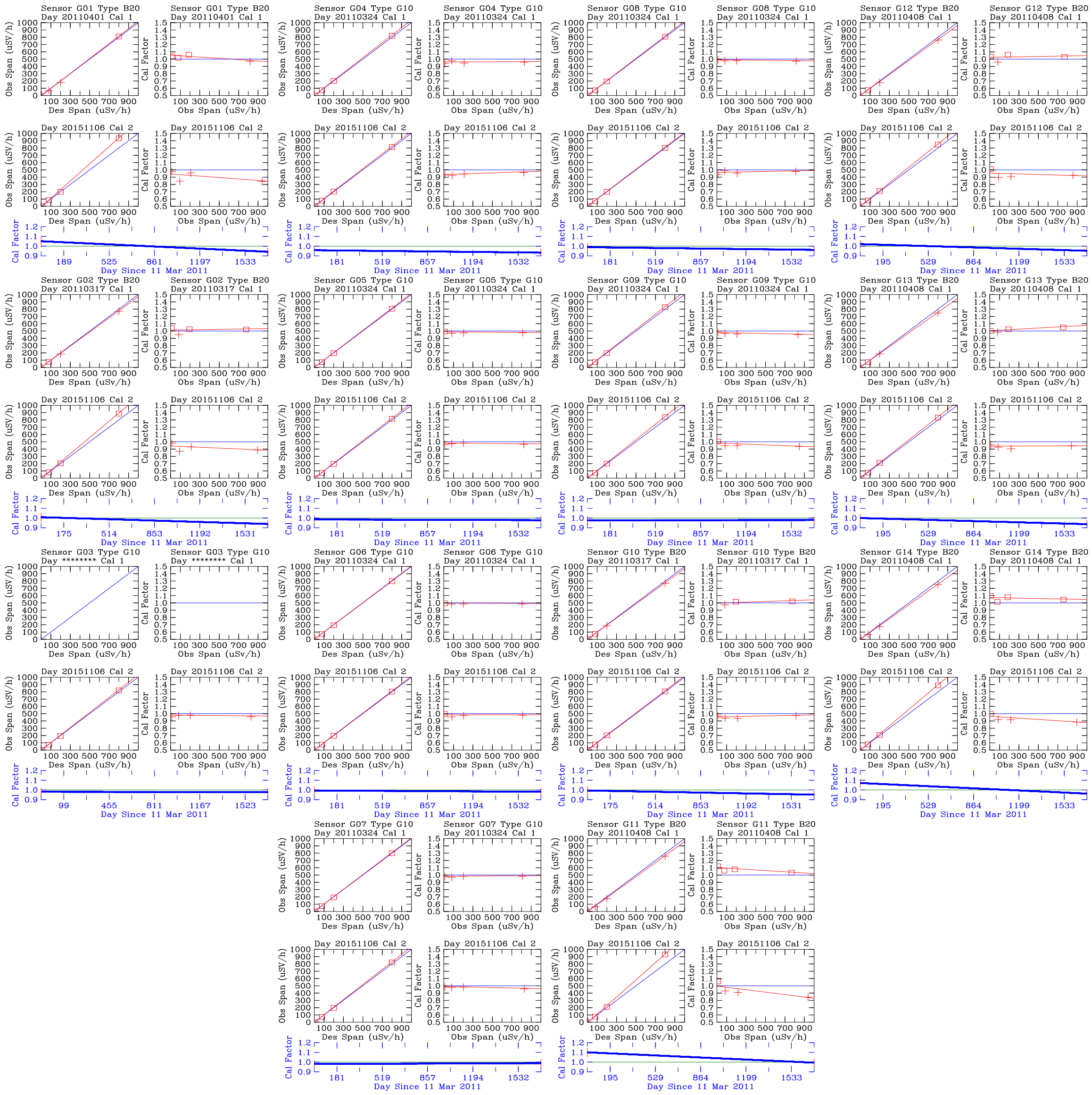

Figure S1. Calibration of fourteen sensors for measurements of air dose rate. Each panel comprises five subpanels. The upper left shows the first comparison between the designated dose rates (Des Span) versus the measured dose rates (Obs Span). The open squares/crosses indicate that the observed values are higher/lower than the designated values. The upper right shows the calibration factors with respect to the designated dose rates. The open squares/crosses indicate that the calibration factors (the ratios of the designated to the observed spans) are greater/lower than one. The lower two sub-panels are the results from the second calibrations. The lowest subpanel shows a linear model of the calibration factors with respect to day.

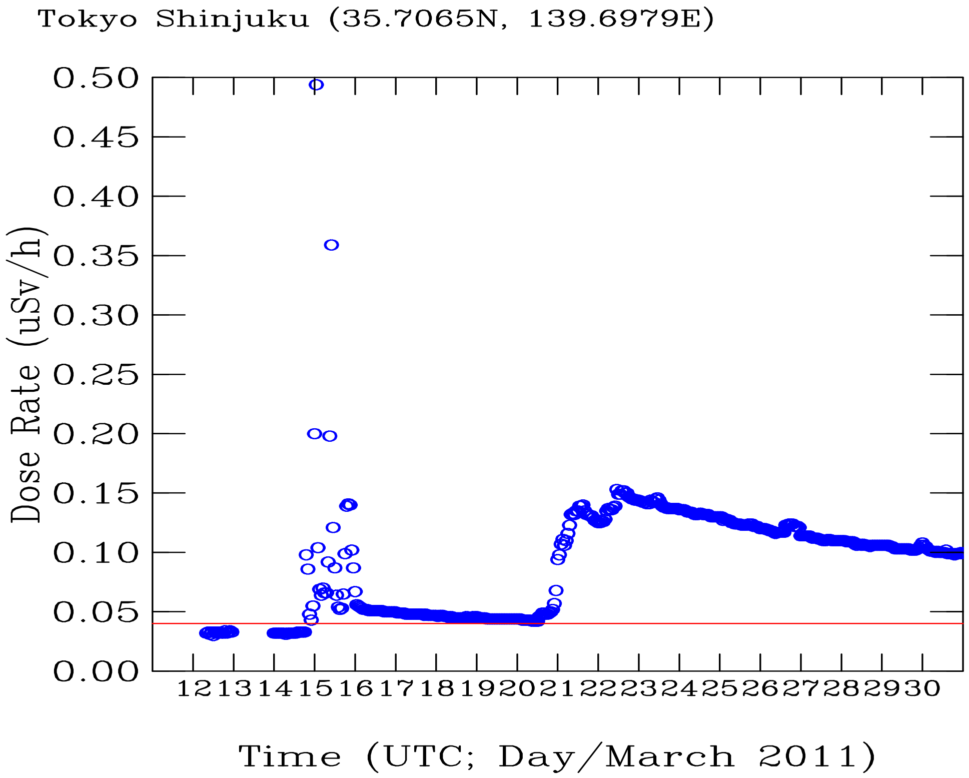

(a)

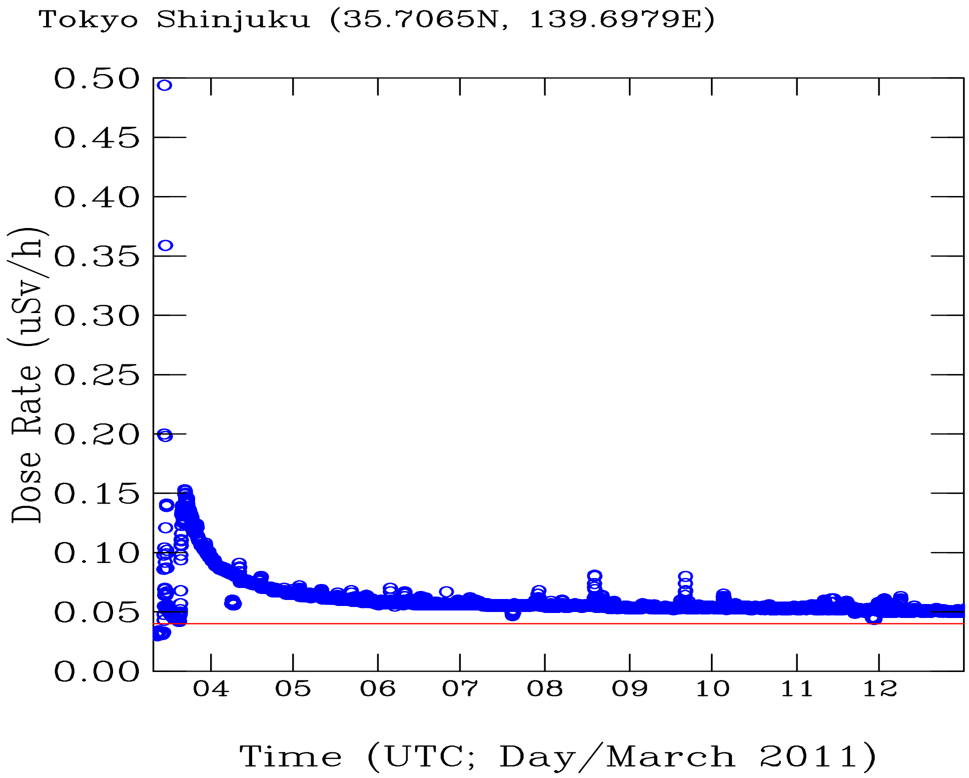

(b)

Figure S2. Time-series measurements of the air dose rates (in the unites of μSv/h) at the Tokyo Shinjuku[51] site (35.7065°N, 139.6979°E). (a) For the period from 11 to 31 March 2011. (b) For the period from 11 March to 31 December 2011. Horizontal red line indicates background level of 0.04 μSv/h.

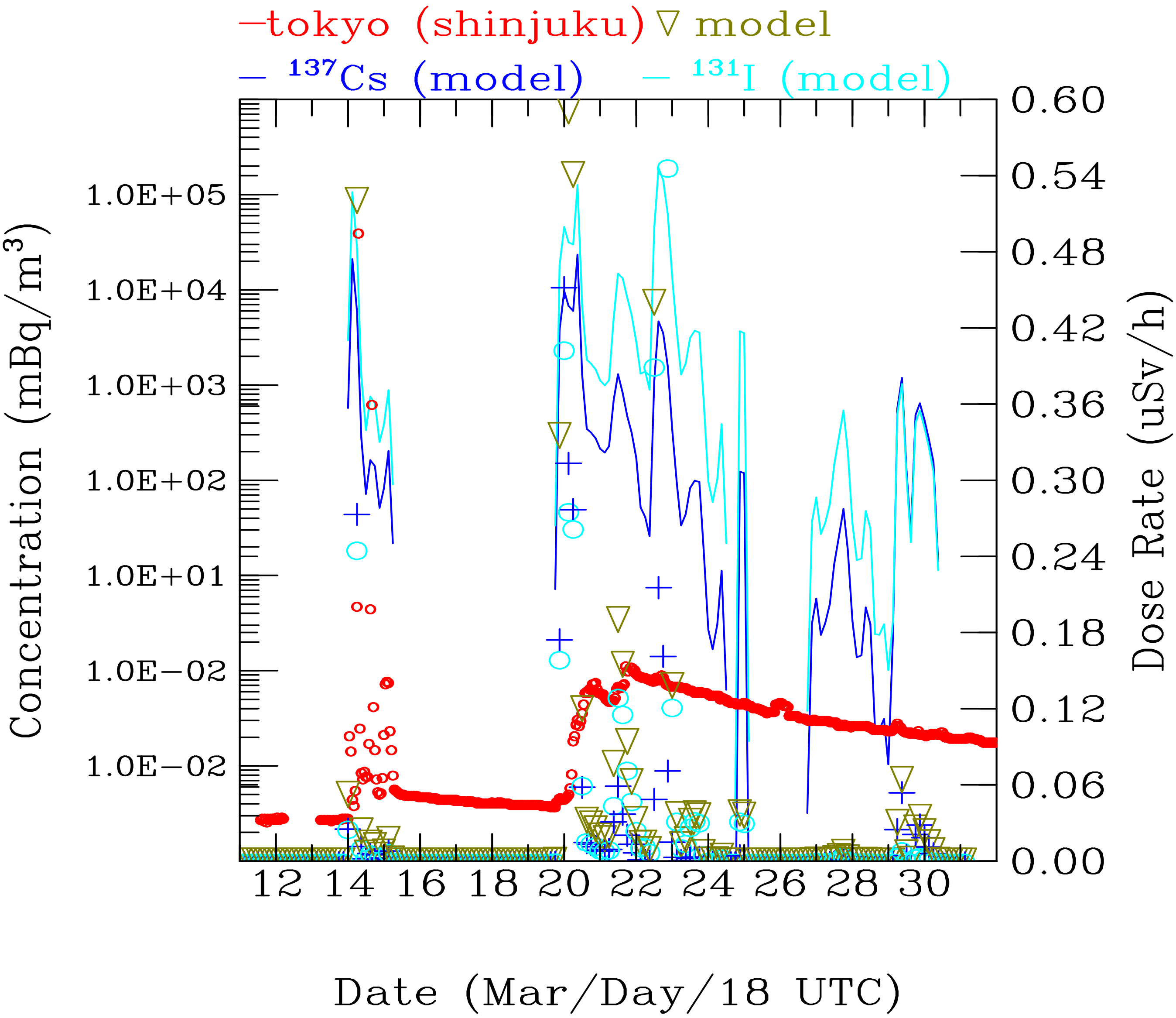

Figure S3 Measurements of the air dose rates (in the unites of μSv/h) and the model simulations of the ^{137}Cs and ^{131}I (in the units of (mBq/m^3, and converted μSv/h, respectively) at the Tokyo Shinjuku[51] site (35.7065°N, 139.6979°E) for the period from 11 to 31 March 2011.

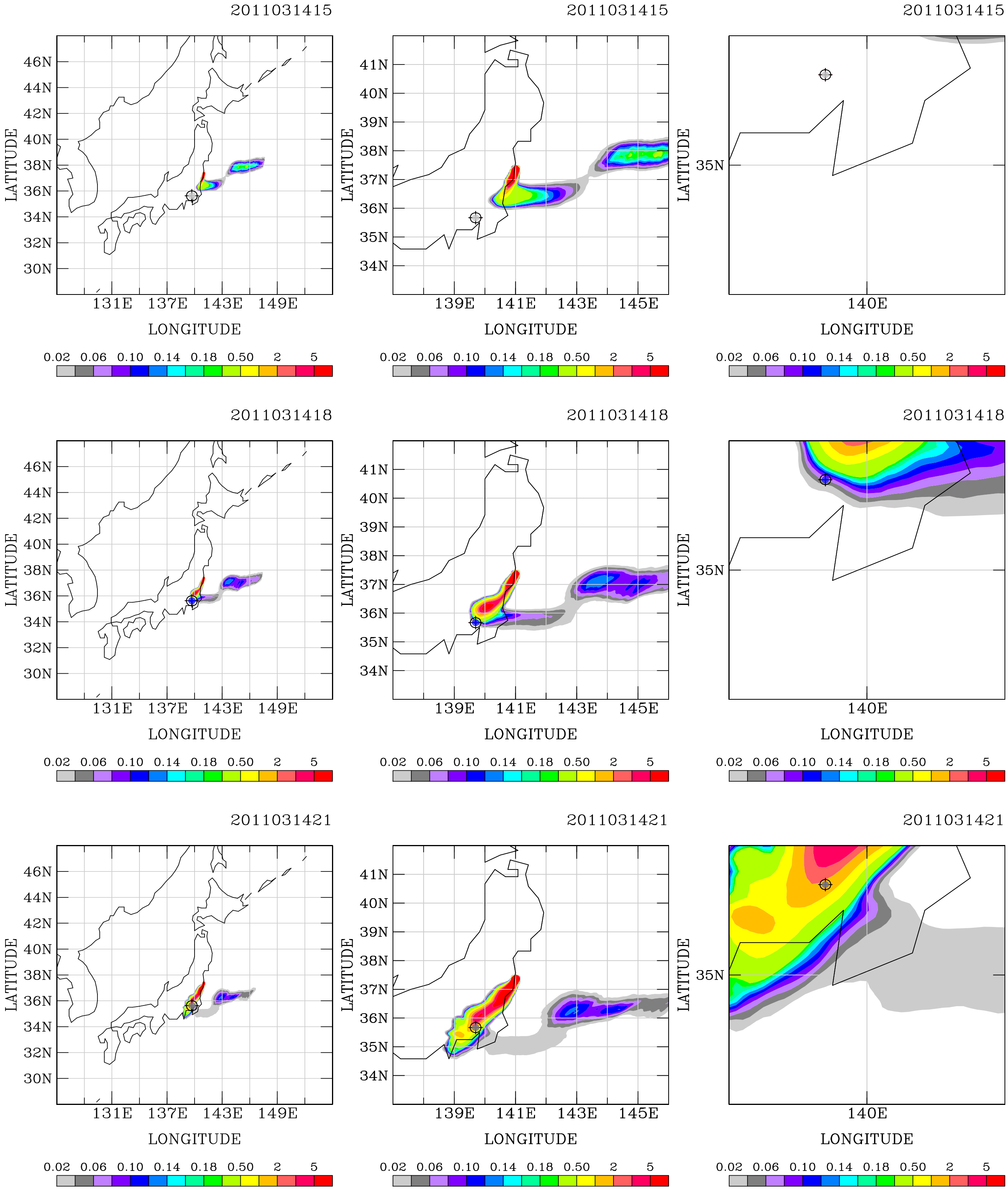

Figure S4 Comparison of the simulated and observed air dose rates on 14 March 2011. Left panels present large-scale, middle panels show magnified , and righ panels show Tokyo Port area views. The upper panels show results at 15 UT, central panels show resluts at 18UT, and the bottom panels show results at 21UT. The measurements at the corresponding times and locations are colored according to the air dose rates and encircled.

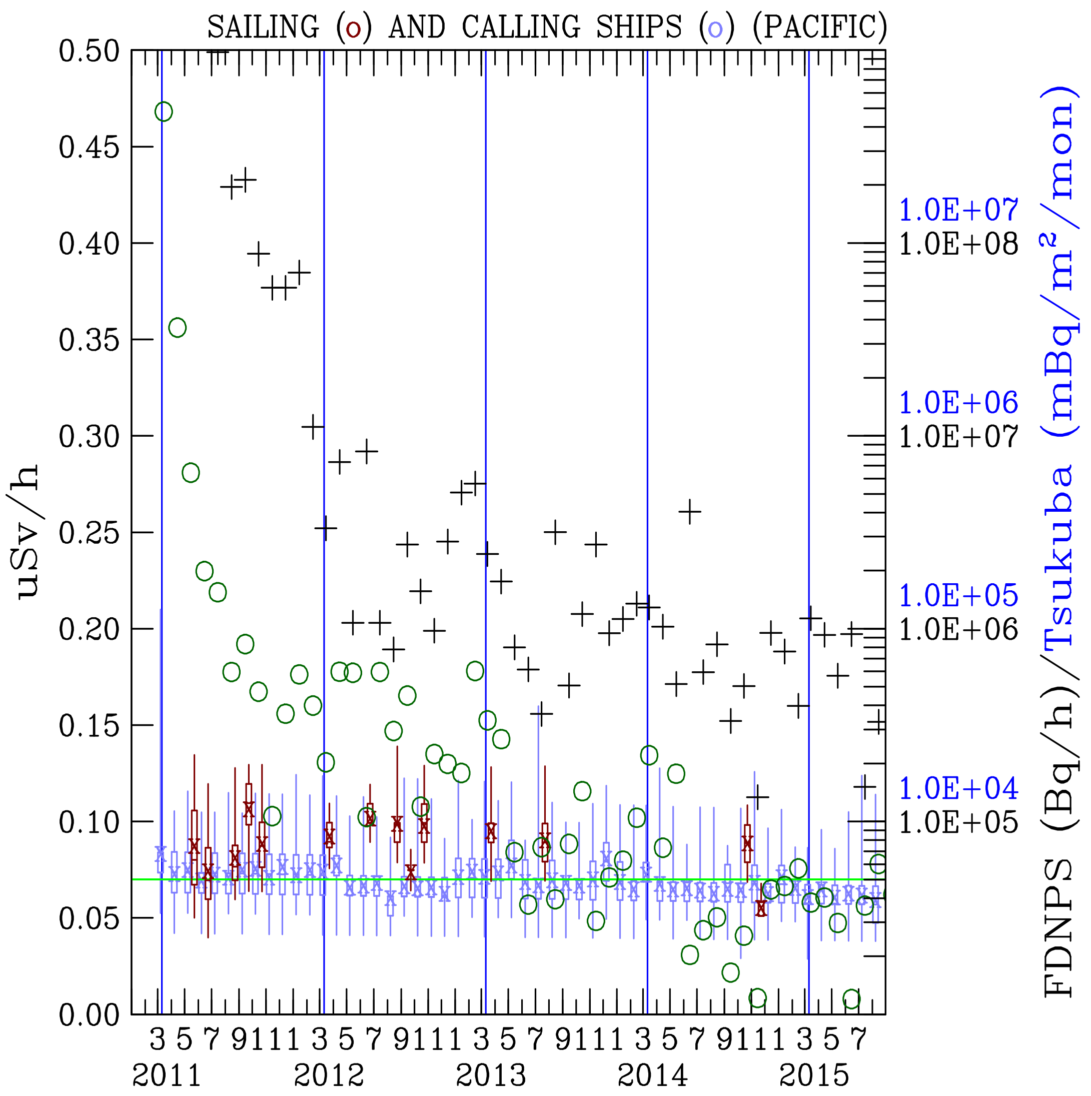

Figure S5. Comparison of the air dose rates measured on the Tokyo Port calling ships (light blue color) and on the Pacific sailing ships (brown color) that had returned to the Tokyo Port. The FDNPS radiocesium emission fluxes are shown as black crosses. The Tsukuba radiocesium deposition fluxes are shown as green open circles.

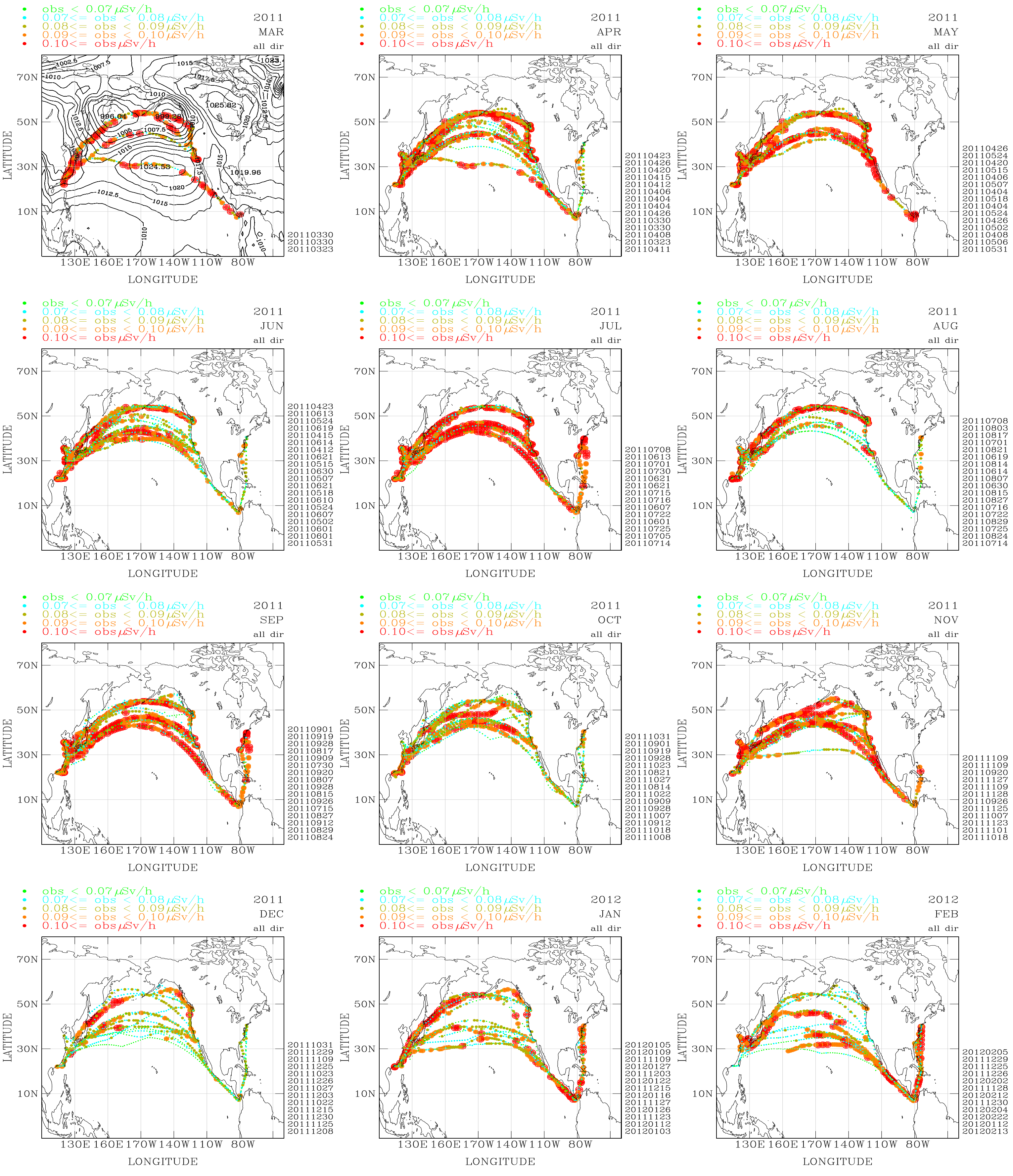

Figure S6. Measurements of the air dose rates over the North Pacific from March 2011 to February 2012. The monthly data are arranged from the top left to righ to the right bottom.

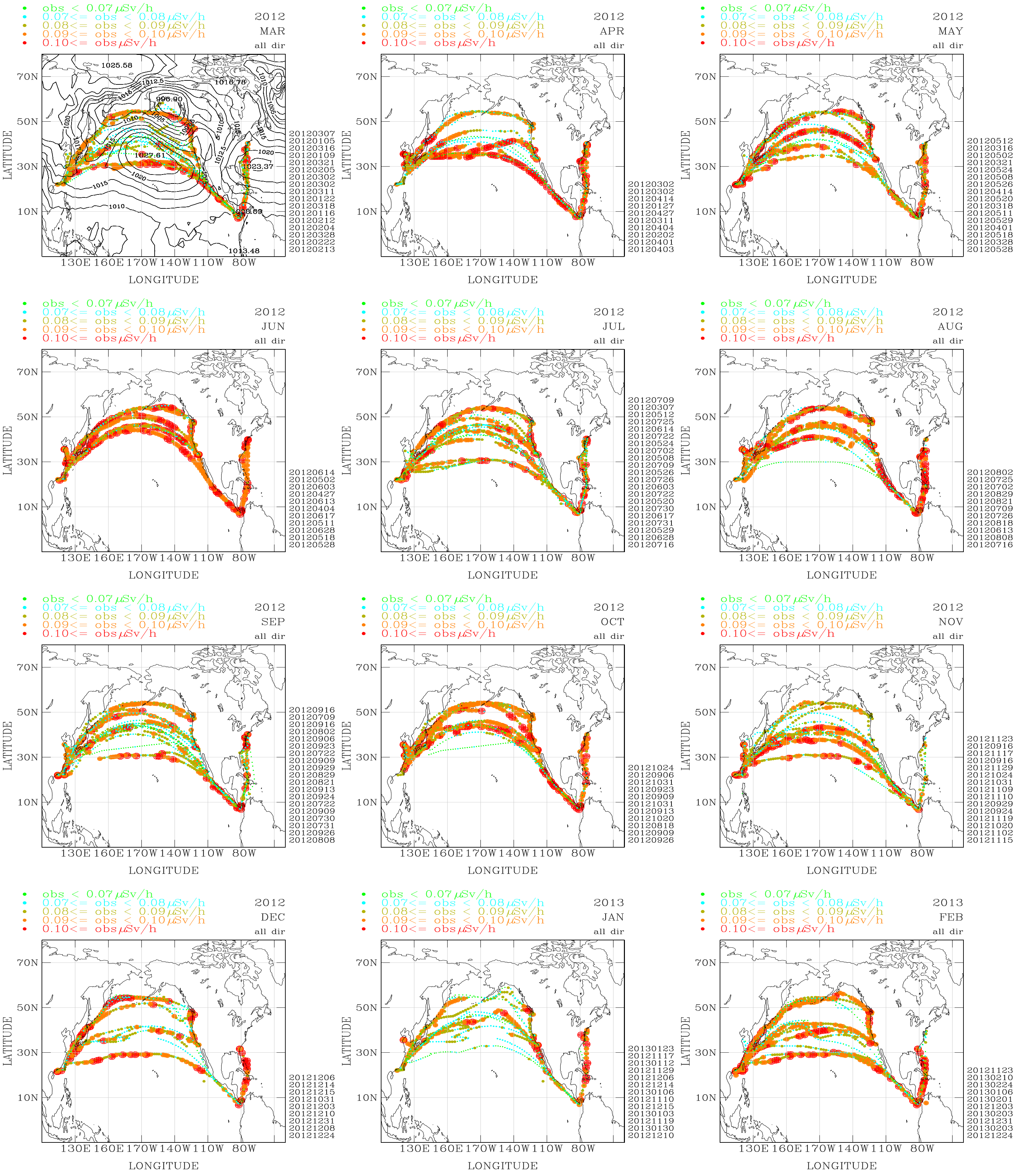

Figure S7. Measurements of the air dose rates over the North Pacific from March 2012 to February 2013. The monthly data are arranged from the top left to righ to the right bottom.

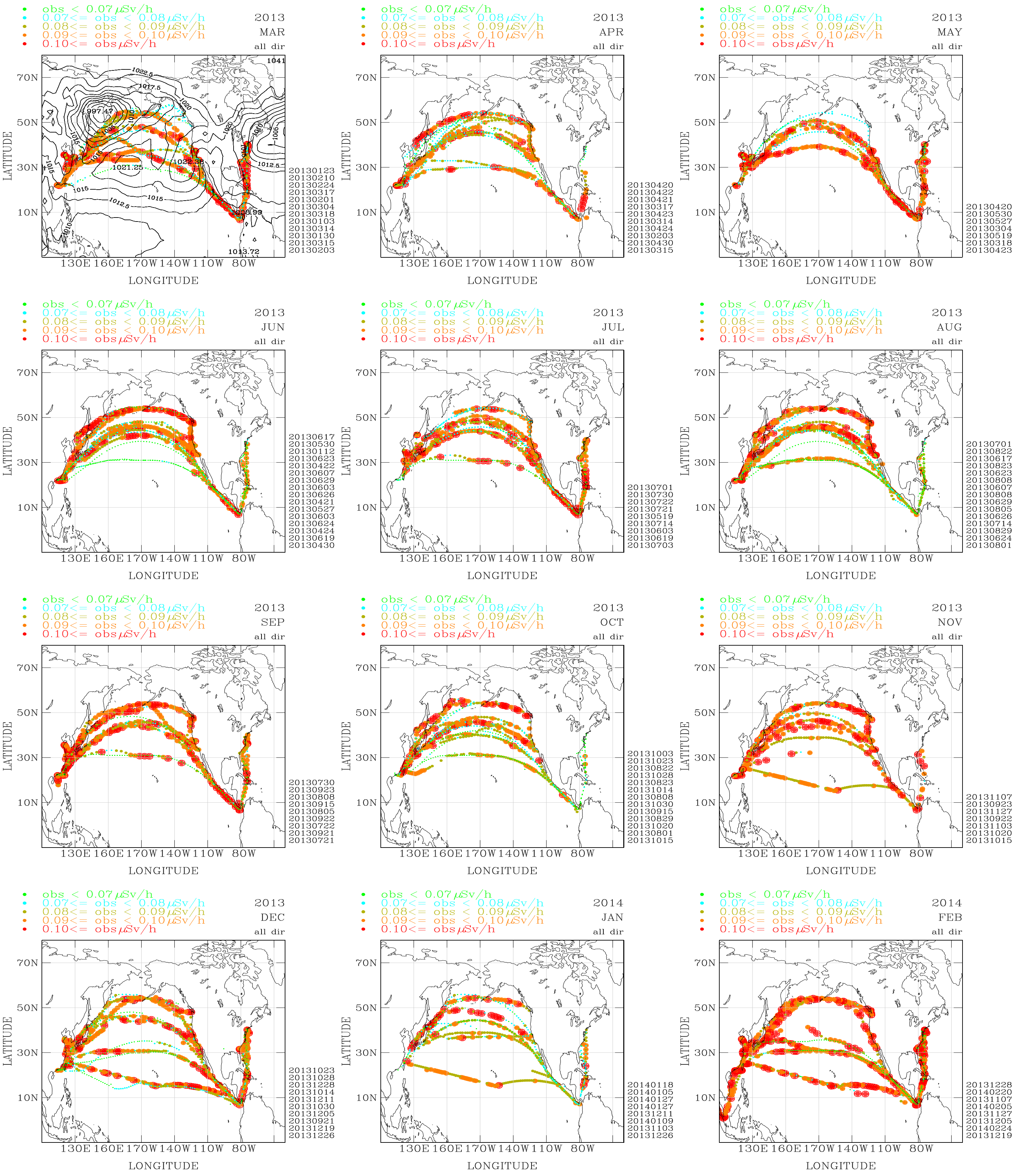

Figure S8. Measurements of the air dose rates over the North Pacific from March 2013 to February 2014. The monthly data are arranged from the top left to righ to the right bottom.

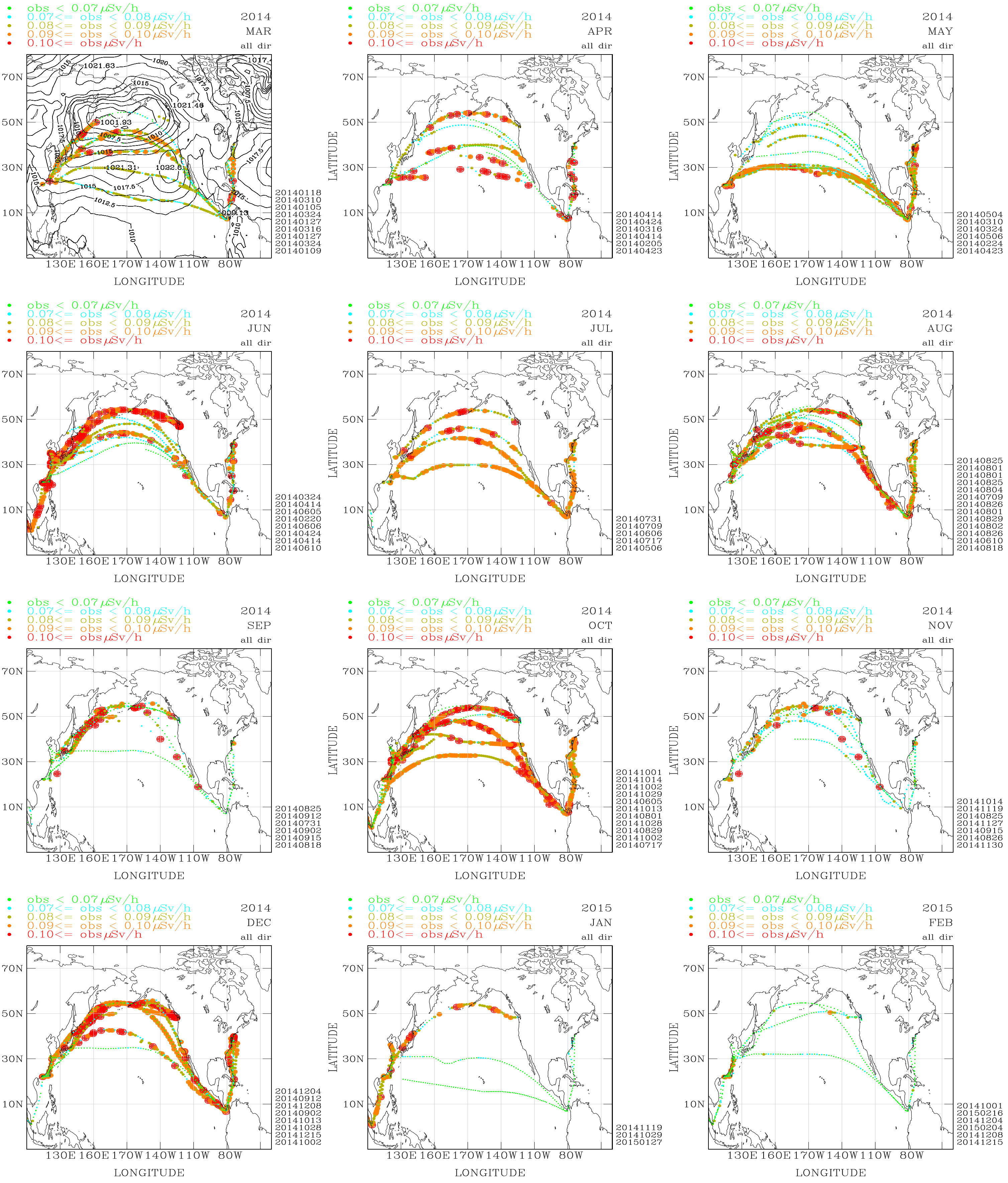

Figure S9. Measurements of the air dose rates over the North Pacific from March 2014 to February 2015. The monthly data are arranged from the top left to righ to the right bottom.

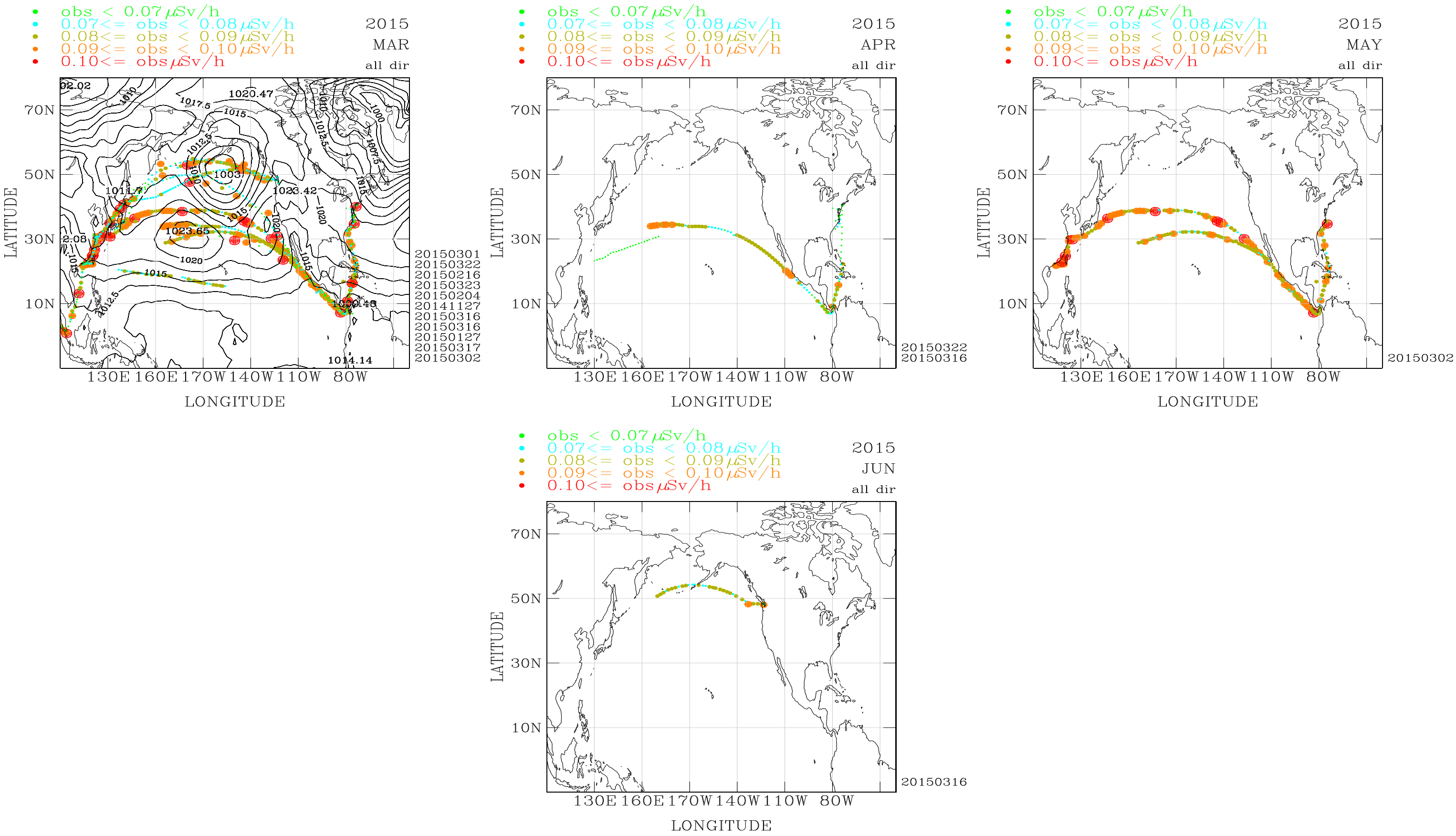

Figure S10. Measurements of the air dose rates over the North Pacific from March to June 2015. The monthly data are arranged from the top left to righ to the bottom.

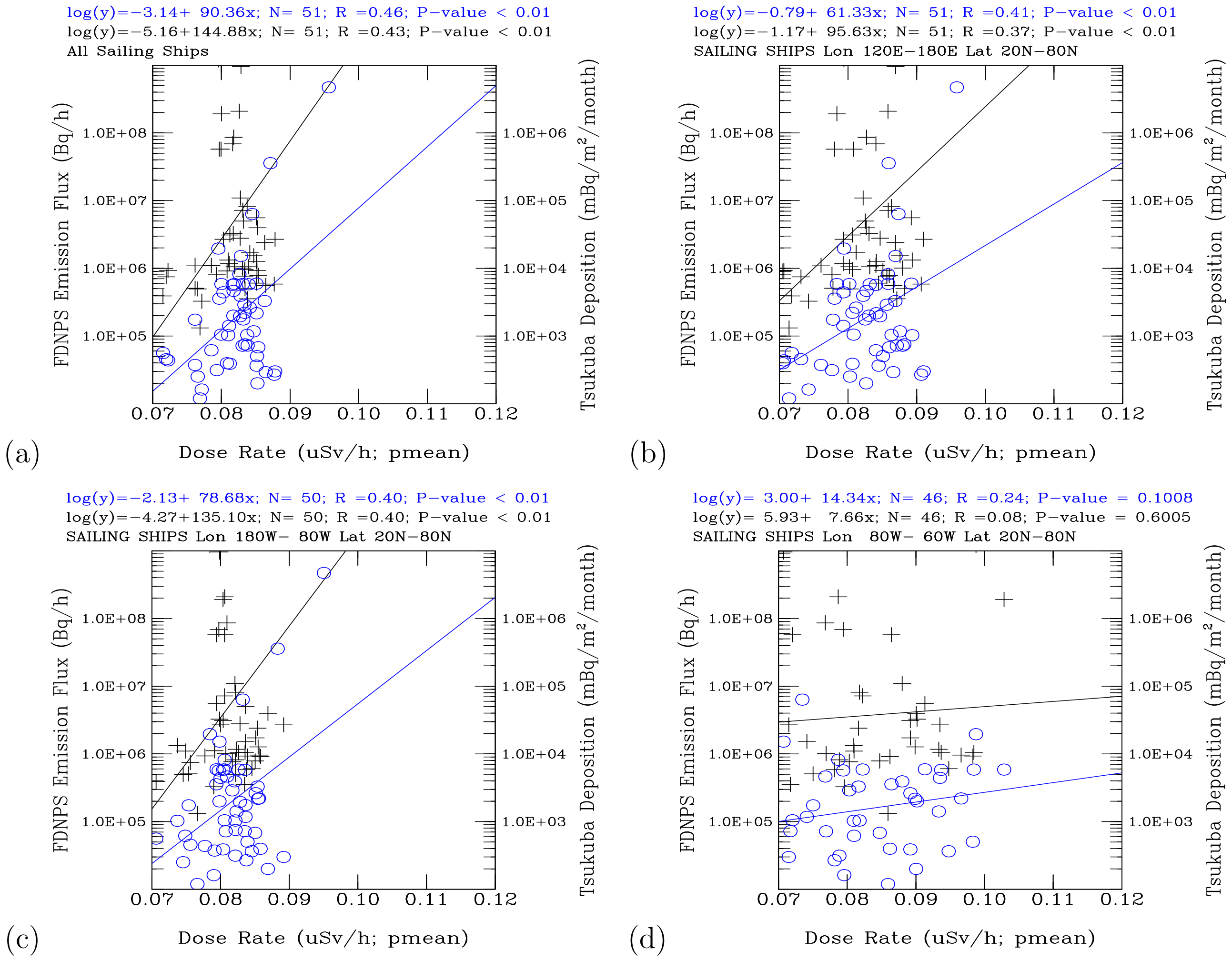

Figure S11. Scatter plot analysis of the FDNPS radiocesium radiocesium emission fluxes (black crosses) and measured Tsukuba radiocesium deposition fluxes (blue circles) versus the monthly mean air dose rates (pmean) on sailing ships. (a) North Pacific Ocean. (b) Northwest Pacific Ocean. (c) Northeast Pacific Ocean. (d) Northwest Atlantic Ocean.

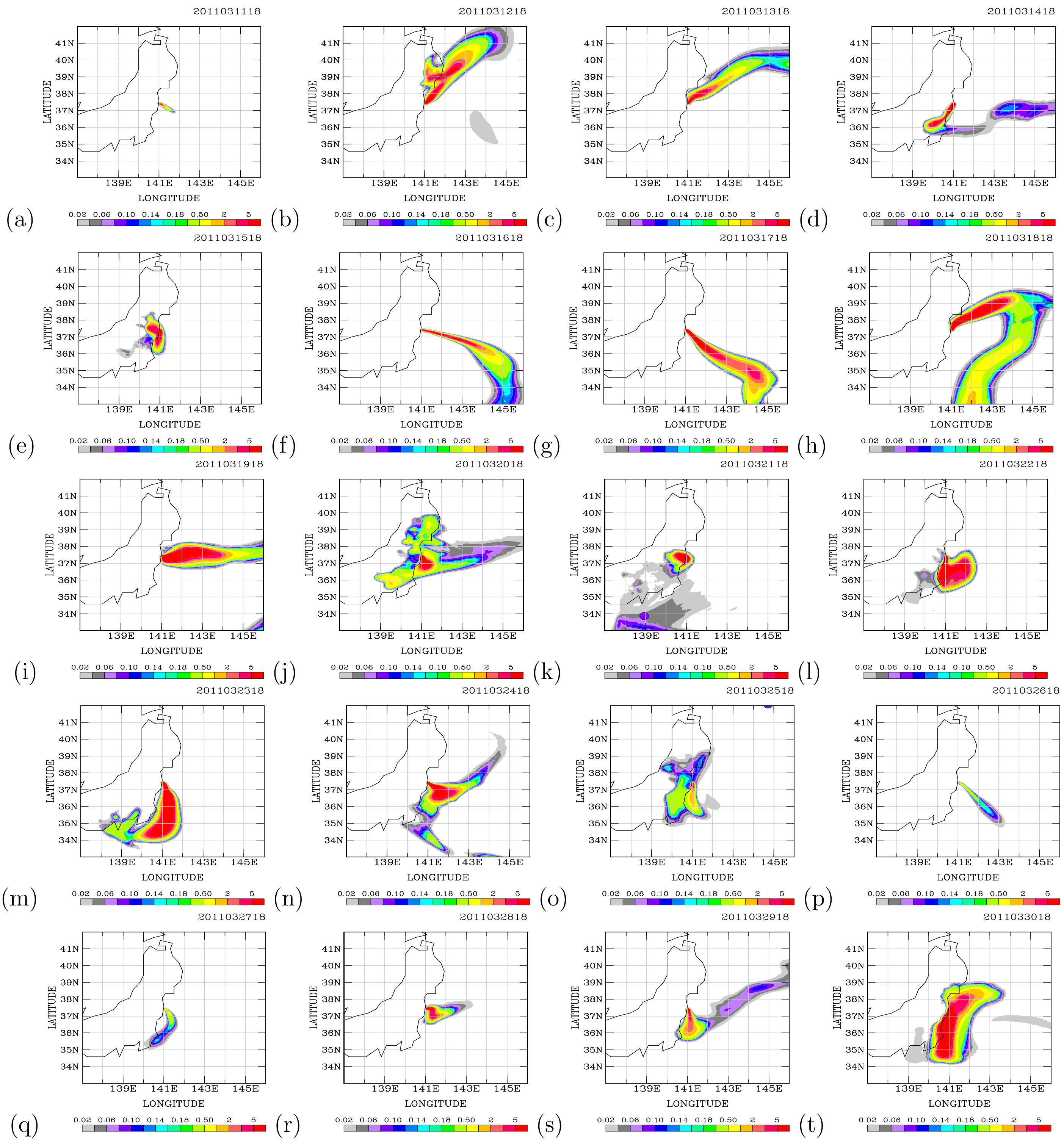

Figure S12. Simulated dispersion of the radionuclides ^{137}Cs and ^{131}I (in the converted units of μSv/h) at 18 UT on each day from 11 to 30 March 2011. Days are ordered from left to right and from top to bottom).

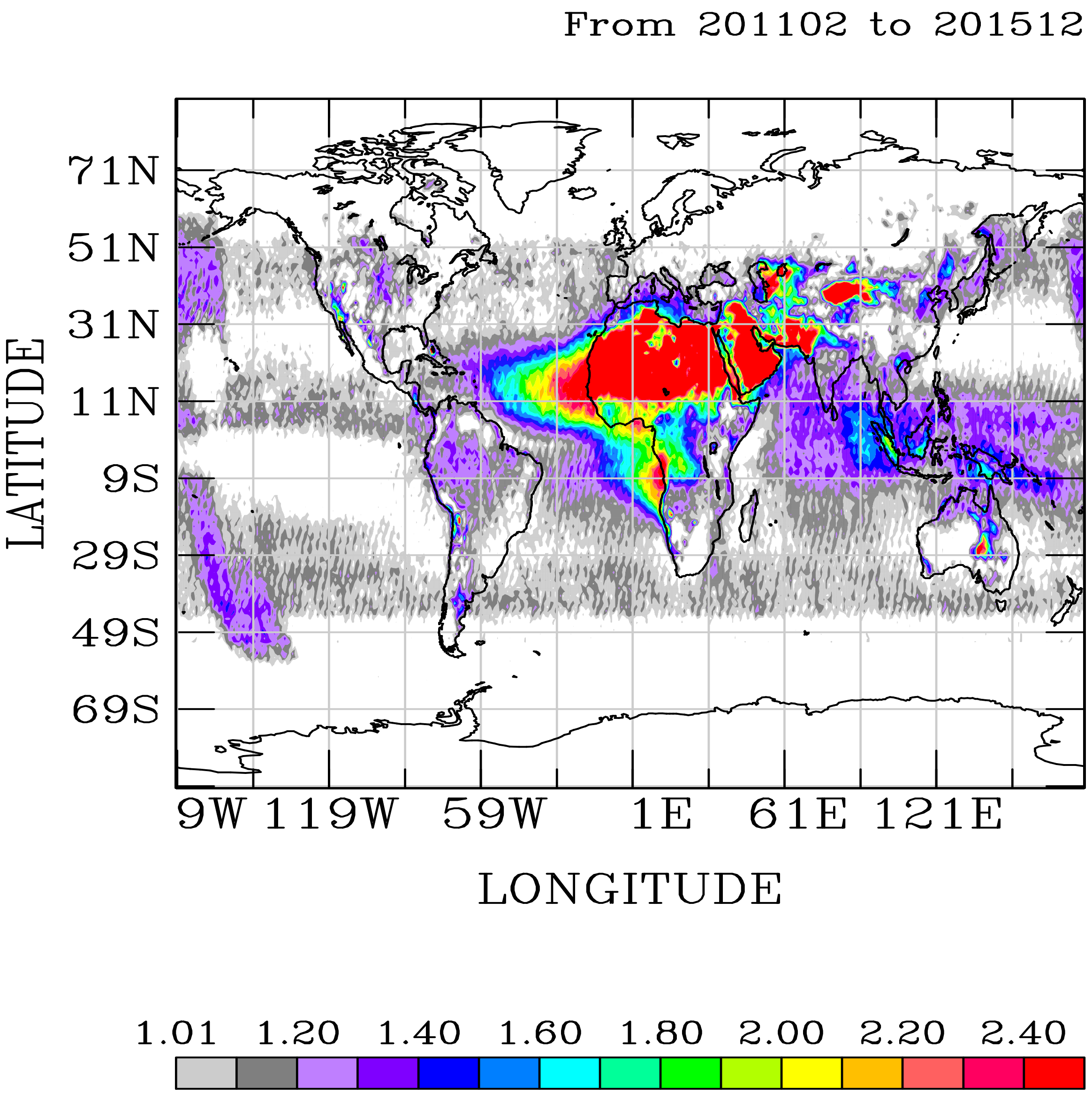

Figure S13. Spatial distribution of the scaled abosrbing aerosol index (AAI) from the OMI satellite. The results are presented on a horizontal resolution of 1 degree longitude-latitude grid system. The values on each grid represents the summation of the monthly AAI, from February 2011 to December 2015, and scaled by the AAI values on a grid covering the Tokyo port area.

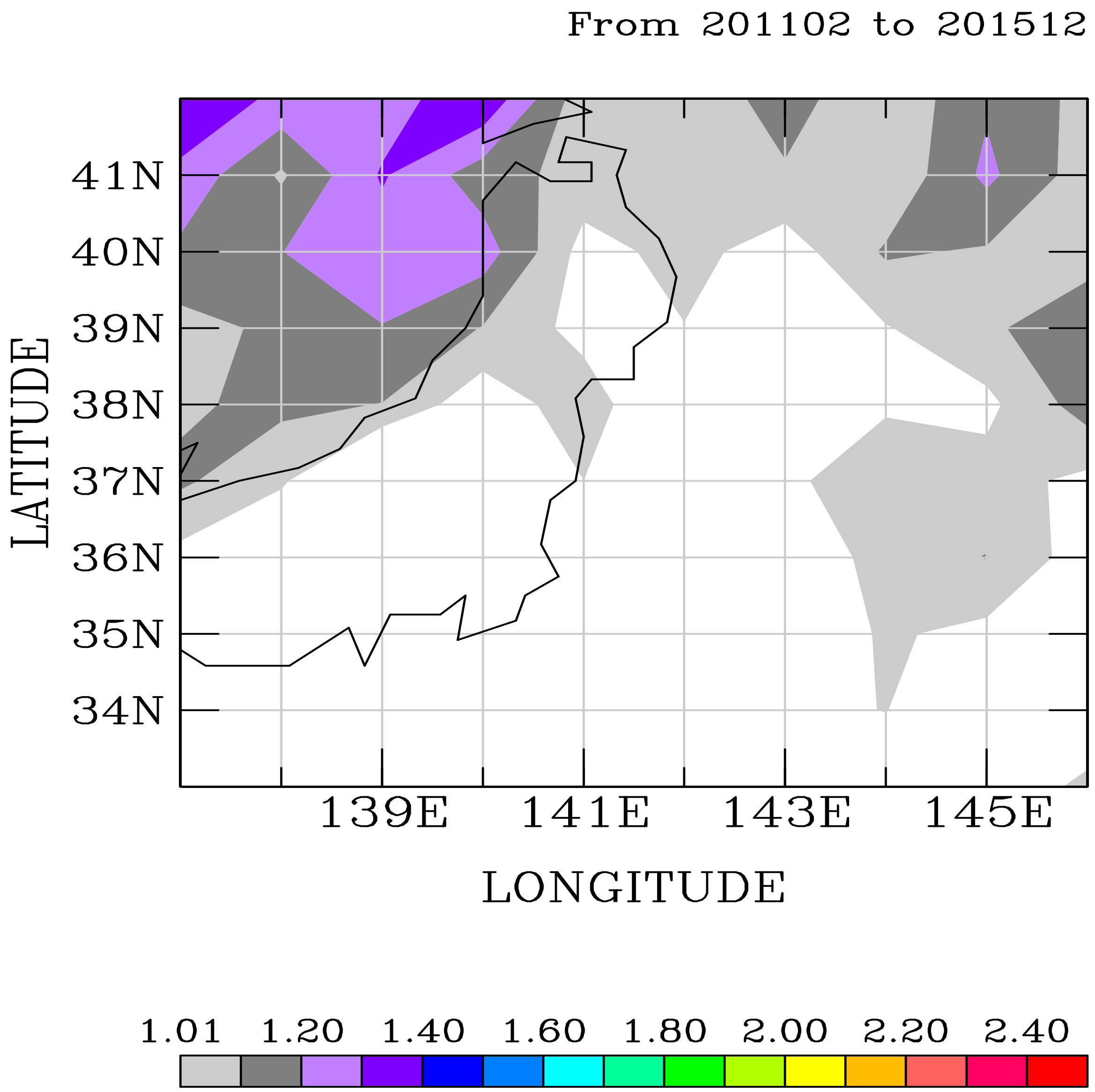

Figure S14. The same as in Figure S15 but for the Japan region, between 137°E and 146°E, and between 33°N and 42°N.

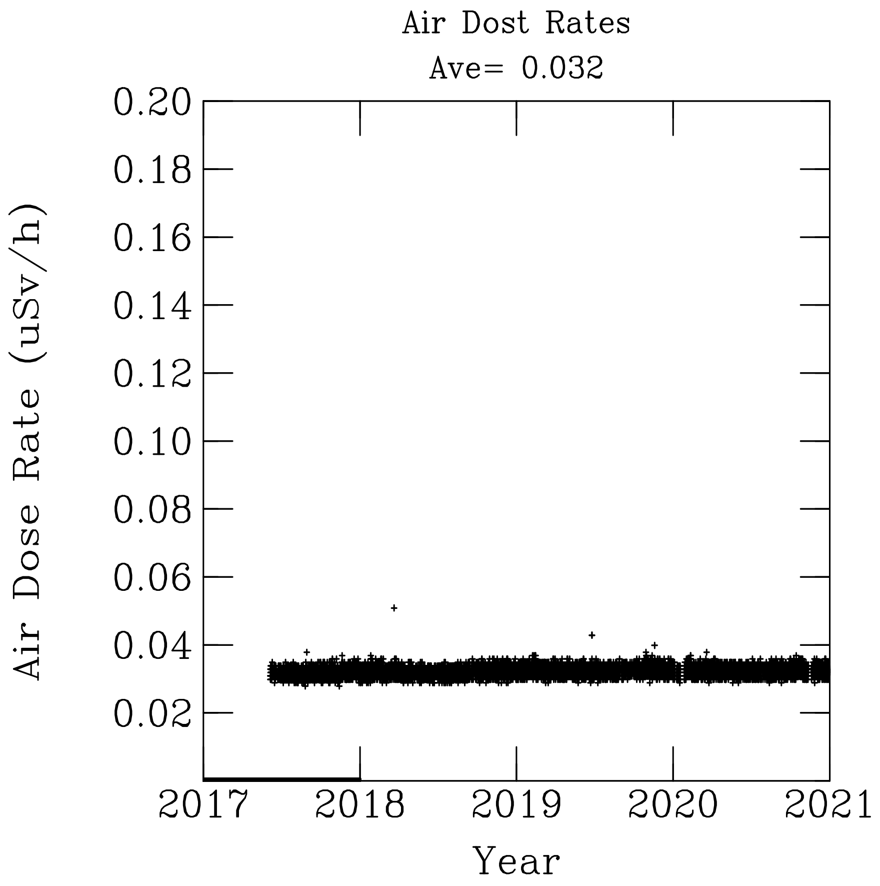

Figure S15. Air dose rates monitored at Hawaii (in the converted units of μSv/h) from 2017 to 2020. The average air dose rates are 0.032 μSv/h.

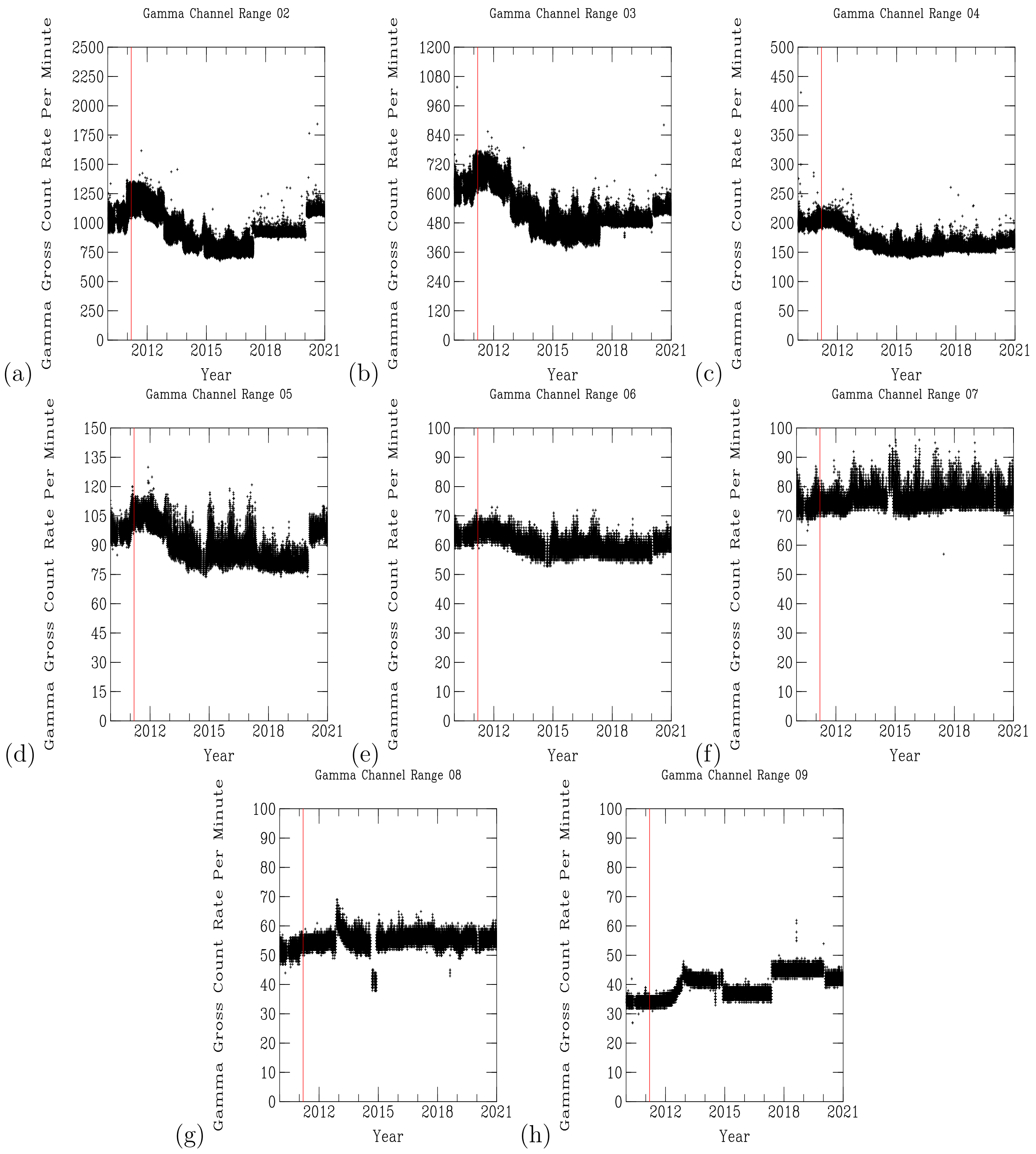

Figure S17. The gamma gross count rates per minute monitored at Hawaii from 2010 to 2020. Monitored results from (a) channel range 02; (b) channel range 03; (c) channel range 04; (d) channel range 05; (e) channel range 06; (f) channel range 07; (g) channel range 08; (h) channel range 09. The vertical red line indicates the time of the FDNPS accident.

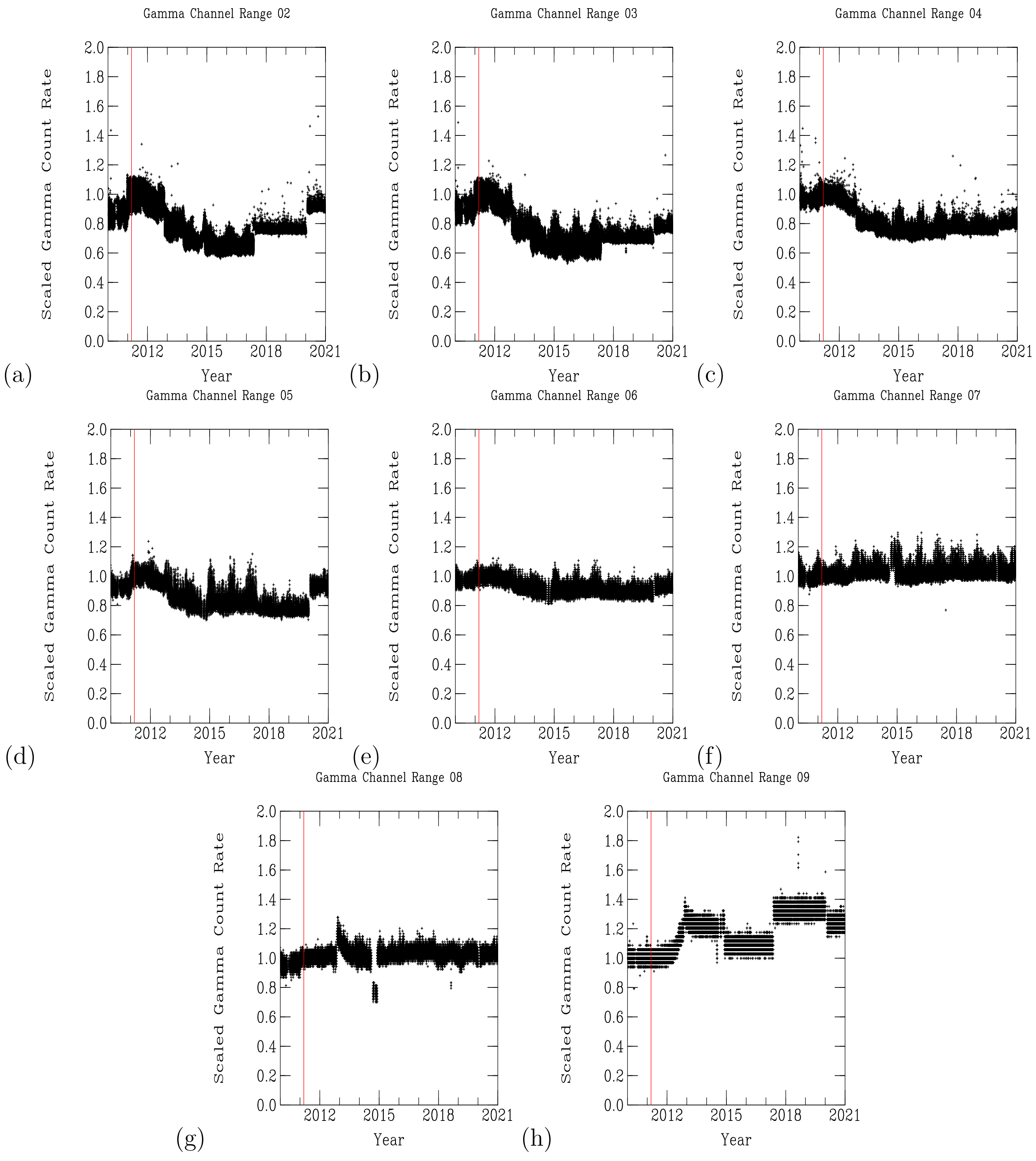

Figure S17. The scaled gamma gross count rates per minute at Hawaii observed from 2010 to 2020. Results are scaled by the 2011 average values. (a) channel range 02; (b) channel range 03; (c) channel range 04; (d) channel range 05; (e) channel range 06; (f) channel range 07; (g) channel range 08; (h) channel range 09. The vertical red line indicates the time of the FDNPS accident.

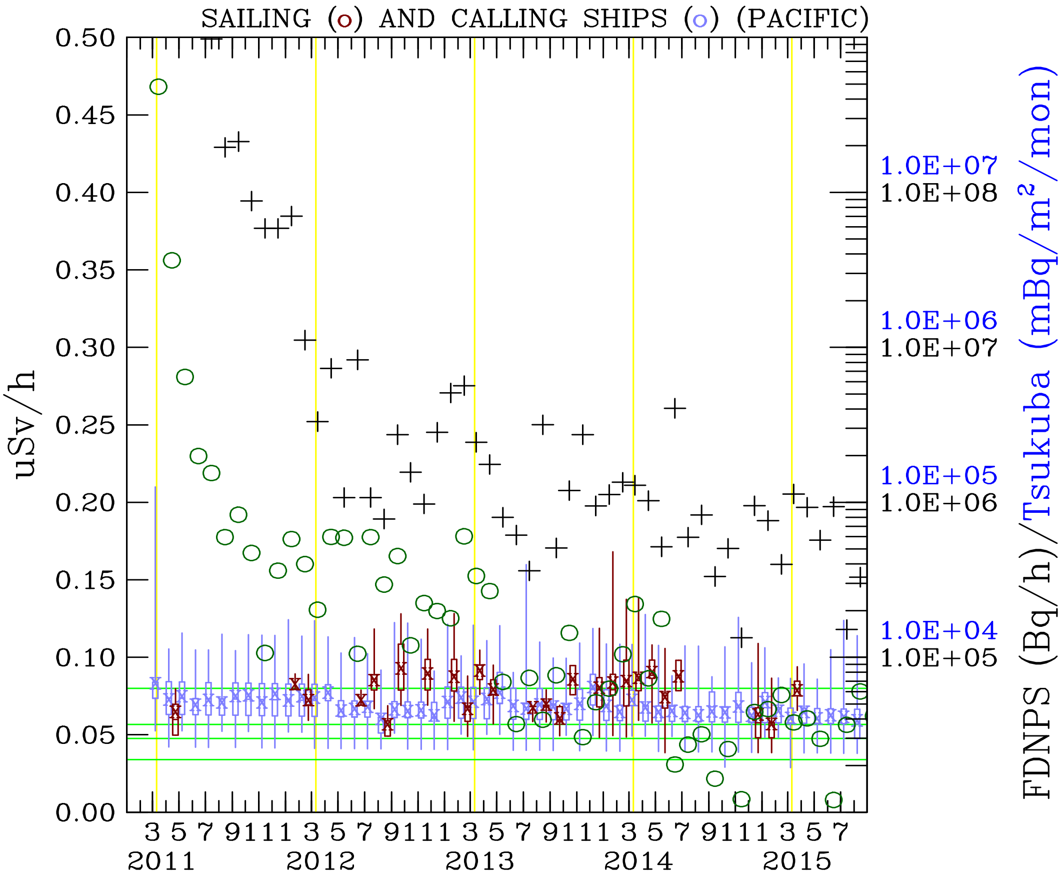

Figure S18. Time-series measurements of the air dose rates (in dark brown color) on the sailing ships in a region containing Hawaii (between 170°W and 140°W, and between longitudes 10°N and 30°N; see also Figure 3). The air dose rates measured on the calling ships of the Tokyo Port are shown in sky blue color for comparison. The lowest horizontal green line shows the 2017-2020 average air dose rates of 0.034 μSv/h measured in Hawaii. The second horizontal green line (from the bottom) shows the estimated air dose rates of 0.048 μSv/h during 2011-2015 (0.034 * 1.4 = 0.048) in Hawaii. The third horizontal green line (from the bottom) shows the estimated air dose rates of 0.057 μSv/h during 2011-2015 in Hawaii (0.034*1/0.60=0.057). Top horizontal green lines shows the estimated air dose rates of 0.080 μSv/h during 2011-2015 (0.034 * 1.4 *1/0.60= 0.080) in Hawaii. Also shown in the figure are the FDNPS emission fluxes (black crosses), and the Tsukuba deposition fluxes (open circles). The vertical yellow lines indicate 11 March in 2011, 2012, 2013, 2014, and 2015.

Tables

Location	Distance to FNPP1	Direction to FNPP1	Dose Rate (μSv/h)	Time	Reference
Minamisoma	25 km	North-northwest	20	12/03/2011	Saunier et al. (2013)
Minamisoma	25 km	North-northwest	20	12/03/2011	Tsuruta et al. (2014)
Minamisoma	25 km	North-northwest	20	20:00, 12/03/2011	Korsakissok et al. (2013)
Tsukuriya Hokota City	139 km	South	< 0.1	14/03/2011	Saunier et al. (2013)
Koriyama	59 km	West	8	15/03/2011	Saunier et al. (2013)
Aizuwakatmasu	98 km	West	2.5	15/03/2011	Saunier et al. (2013)
Iitate	39 km	Northwest	45	15/03/2011	Saunier et al. (2013)
Utsunomiya	140 km	South	1.3	15/03/2011	Saunier et al. (2013)
Ishikawa	126 km	South	1.5	15/03/2011	Saunier et al. (2013)
Minamiaizu	114 km	West	0.9	15/03/2011	Saunier et al. (2013)
Shirakawa	80 km	West	7.5	15/03/2011	Saunier et al. (2013)
Daini (FNPP2)	15 km	South	94	00:00, 15/03/2011	Korsakissok et al. (2013)
Iwaki	40 km	South-southwest	23.7	01:00, 15/03/2011	Korsakissok et al. (2013)
Tsukuriya Hokota City	139 km	South	4	07:00, 15/03/2011	Saunier et al. (2013)
Kawauchi	22 km	Southwest	11.5	11:00, 15/03/2011	Korsakissok et al. (2013)
Koriyama	60 km	West	6	14:00, 15/03/2011	Korsakissok et al. (2013)
Iitate	40 km	Northwest	39.50	15:00, 15/03/2011	Korsakissok et al. (2013)
Fukushima	61 km	Northwest	24	16:00, 15/03/2011	Korsakissok et al. (2013)
Tsukuba	176 km	South-southwest	1	15/03/2011	Tsuruta et al. (2014)
Momijiyama	25 km	Northwest	16	15 – 16/03/2011	Tsuruta et al. (2014)
Tsukuriya Hokota City	139 km	South	1.5	06:00, 16/03/2011	Saunier et al. (2013)
Yamamoto	126 km	North	1.6	16/03/2011	Saunier et al. (2013)
Yamagata	111 km	North	< 0.1	16/03/2011	Saunier et al. (2013)
Minamisoma	25 km	North-northwest	6-8	18 – 21/03/2011	Tsuruta et al. (2014)
Fukushima Tunnel	61 km	Northwest	0.4	19/03/2011	Kubota et al. (2013)
Fukushima Tunnel	61 km	Northwest	1.0	20/03/2011	Kubota et al. (2013)
Tsukuriya Hokota City	139 km	South	0.8	12:00, 20/03/2011	Saunier et al. (2013)
Nihonmatsu City	56 km	West-northwest	5.8	20/03/2011	Kubota et al. (2013)
Fukushima Tunnel	61 km	Northwest	2.0	21/03/2011	Kubota et al. (2013)
Fukushima City	61 km	Northwest	2-9	19 – 22/03/2011	Kubota et al. (2013)
Tohoku Expressway	64 km	Northwest	2-6	19 – 22/03/2011	Kubota et al. (2013)
Banetsu Expressway	64 km	West	1.5-3	19 – 22/03/2011	Kubota et al. (2013)
Samegawa Village	64 km	Southwest	1	19 – 22/03/2011	Kubota et al. (2013)
Momijiyama	25 km	Northwest	7-9	20 – 21/03/2011	Tsuruta et al. (2014)
Tsukuba	176 km	South-southwest	0.2-0.4	20 – 21/03/2011	Tsuruta et al. (2014)
Tsukuriya Hokota City	139 km	South	2.2	06:00, 21/03/2011	Saunier et al. (2013)
Nihonmatsu City	56 km	West-northwest	0.17	09/2011	Fujimura et al. (2017)
Nihonmatsu City	56 km	West-northwest	0.17	2012	Fujimura et al. (2017)
Nihonmatsu City	56 km	West-northwest	0.11	2013	Fujimura et al. (2017)
Nihonmatsu City	56 km	West-northwest	0.07	03 – 05/2014	Fujimura et al. (2017)

Table S1. List of published measurements of the air dose rates.

Year	Month	Day	Hour	Model ^{137}Cs	Model ^{131}I	Model ^{137}Cs $+^{131}$ I	Obs Min	Obs Max	Obs Mean
2011	3	22	06	0.0442	0.1161	0.1603	0.1050	0.2100	0.1573
2011	3	23	02	0.0030	0.0225	0.0255	0.0630	0.1050	0.0820
2011	3	25	12	0.0000	0.0000	0.0000	0.0520	0.1150	0.0820
2011	3	26	10	0.0000	0.0000	0.0000	0.0630	0.0840	0.0750
2011	3	27	23	0.0000	0.0000	0.0000	0.0630	0.0840	0.0768
2011	3	28	04	0.0007	0.0015	0.0023	0.0730	0.0840	0.0748
2011	3	28	10	0.0021	0.0043	0.0064	0.0730	0.0840	0.0803
2011	3	29	10	0.0000	0.0000	0.0000	0.0630	0.0940	0.0768
2011	3	31	01	0.0131	0.0018	0.0150	0.0630	0.1050	0.0787

Table S2. List of simulations and measurements on the Tokyo Port calling ships (in units of μSv/h).
