# Supplementary material for: Impacts on air dose rates after the Fukushima accident over the North Pacific from 19 March 2011 to 2 September 2015
Source: PLoS One. 2022 Aug 24;17(8):e0272937. doi: 10.1371/journal.pone.0272937 (PMC9401177; doi:10.1371/journal.pone.0272937)
Supplement: Attachment — Submitted filename: Response to Reviewers.pdf [file pone.0272937.s002.pdf]

Response to reviewers’ comments. Manuscript Number PONE-D-21-14834

Impacts on air dose rates after the Fukushima accident over the North Pacific from 19 March 2011 to 2 September 2015

By Wang et al.

We are very grateful indeed to the reviewers, the editor, and the editorial office for very encouraging and insightful comments that have significantly increased the clarity of the manuscript. We have revised the manuscript following the comments. A detailed point to point response to each comment is given below.

Please include the following items when submitting your revised manuscript:
* A rebuttal letter that responds to each point raised by the academic editor and reviewer(s). You should upload this letter as a separate file labeled 'Response to Reviewers'.

Reply. We have uploaded the Response to Reviewers.

* A marked-up copy of your manuscript that highlights changes made to the original version. You should upload this as a separate file labeled 'Revised Manuscript with Track Changes'.

Reply. We have uploaded a separated file, labeled “Revised_Manuscript_with_Track_Changes.pdf”.

* An unmarked version of your revised paper without tracked changes. You should upload this as a separate file labeled 'Manuscript'.

Reply. We have uploaded an unmarked version of the revised manuscript.

Journal Requirements:

When submitting your revision, we need you to address these additional requirements.

1. Please ensure that your manuscript meets PLOS ONE's style requirements,

including
those for file naming. The PLOS ONE style templates can be found at https://journals.plos.org/plosone/s/file?id=wjVg/PLOSOne_formatting_sample_main_body.pdf and https://journals.plos.org/plosone/s/file?id=ba62/PLOSOne_formatting_sample_title_authors_affiliations.pdf

Reply. We have reformatted the manuscript following the PLOS LaTex template.

2. Please update your submission to use the PLOS LaTeX template. The template and more information on our requirements for LaTeX submissions can be found at http://journals.plos.org/plosone/s/latex.

Reply. We have submitted the revised manuscript using the PLOS LaTex template.

3. We note that the grant information you provided in the ‘Funding Information’ and ‘Financial Disclosure’ sections do not match. When you resubmit, please ensure that you provide the correct grant numbers for the awards you received for your study in the ‘Funding Information’ section.

Reply. We have provided grant number in the Acknowledgements.

4. Thank you for stating the following financial disclosure: "KYW is funded by the Ministry of Science and Technology (www.most.gov.tw; 107-2111-M-008-027-). PN and HC are supported by the the IAGOS project (www.iagos.org)."

Reply. Thank you very much for your confirmation.

Please state what role the funders took in the study. If the funders had no role, please state: "The funders had no role in study design, data collection and analysis, decision to publish, or preparation of the manuscript." If this statement is not correct you must amend it as needed. Please include this amended Role of Funder statement in your cover letter; we will change the online submission form on your behalf.

Reply. We have included this Role of Funder statement in the cover letter.

5. We note that you have stated that you will provide repository information for your data at acceptance. Should your manuscript be accepted for publication, we will hold

it until you provide the relevant accession numbers or DOIs necessary to access your data. If you wish to make changes to your Data Availability statement, please describe these changes in your cover letter and we will update your Data Availability statement to reflect the information you provide.

Reply. We have described in the manuscript that all the data of air dose rates measurements reported in this work are openly available at http://doi.org/10.5281/zenodo.3563214.

6. We note that Figure 1 in your submission contain satellite images which may be copyrighted. All PLOS content is published under the Creative Commons Attribution License (CC BY 4.0), which means that the manuscript, images, and Supporting Information files will be freely available online, and any third party is permitted to access, download, copy, distribute, and use these materials in any way, even commercially, with proper attribution. For these reasons, we cannot publish previously copyrighted maps or satellite images created using proprietary data, such as Google software (Google Maps, Street View, and Earth). For more information, see our copyright guidelines: http://journals.plos.org/plosone/s/licenses-and-copyright.

We require you to either (1) present written permission from the copyright holder to publish these figures specifically under the CC BY 4.0 license, or (2) remove the figures from your submission:

Reply. We have replaced the satellite image used in Fig. 1 with a satellite image from CSRSR. We have obtained a written permission from the Director of CSRSR for using this satellite in the manuscript.

a. You may seek permission from the original copyright holder of Figure 1 to publish the content specifically under the CC BY 4.0 license.

We recommend that you contact the original copyright holder with the Content Permission Form (http://journals.plos.org/plosone/s/file?id=7c09/content-permission-form.pdf) and the following text: “I request permission for the open-access journal PLOS ONE to publish XXX under the Creative Commons Attribution License (CCAL) CC BY 4.0 (http://creativecommons.org/licenses/by/4.0/). Please be aware that this license allows unrestricted use and distribution, even commercially, by third parties. Please reply and provide explicit written permission to publish XXX under a CC BY license

and complete the attached form.”

Please upload the completed Content Permission Form or other proof of granted permissions as an "Other" file with your submission.

Reply. We have uploaded the completed Content Permission Form signed by CSRSR.

In the figure caption of the copyrighted figure, please include the following text: “Reprinted from [ref] under a CC BY license, with permission from [name of publisher], original copyright [original copyright year].”

Reply. We have included the copyright text in the Figure 1 caption.

7. Please include captions for your Supporting Information files at the end of your manuscript, and update any in-text citations to match accordingly. Please see our Supporting Information guidelines for more information: http://journals.plos.org/plosone/s/supporting-information.

Reply. We have included captions of Supporting Information files at the end of the manuscript.

Additional Editor Comments (if provided):

Please add a thorough discussion of the suitability of the GM counter for the measurements reported in your manuscript.

Reply. We have included a thorough discussion of the suitability of the GM counter for the measurements reported in this work in the section Discussion.

Include any technical data that support the use of the GM counter for this work, and also cite and discuss other examples in which comparable measurements have been made with this instrument.

Reply. The GM counters RadEye B20 and G10 were used in this work. Both GM counters have been extensively used in published works related to the measurements of air dose rates in places associated with tests of nuclear weapons, accidents from nuclear power plants, medical imaging processes with patients

injected with nuclear materials, robotic exploration of nuclear environment, radioactivity safety training, etc.

We have included technical data from above examples that support the use of the GM counter for this work. We have cited and discussed above examples in which comparable measurements have made with the GM counter used in this work.

Reviewer #1: General comments
The authors present the first continuous measurements of air dose rates in the ocean atmosphere after a major accident at a nuclear power plant. The data is considered to be very valuable. The amount of data is shown by many figures, but it was difficult to read many supplementary figures, especially in the discussion. I think it would have been easier to understand the discussions if they were presented together with the results. In terms of the suspension, I think it is necessary to discuss whether it is the effect of the Fukushima accident or the effect from the continent due to atmospheric nuclear weapons tests I believe that this paper is suitable for the publication in PLOS ONE after minor revisions.

Reply. We are very grateful to the reviewer for very insightful comments that have significantly increased the clarity of the manuscript. We have followed reviewer's comments to revised the manuscript. In the following, we provide point-to-point responses to reviewer's comments.

Specific comments
L36 The PGGM project needs to be cited here.

Reply. We have included a citation to the PGGM project.

Figure 1 and 2; The earth quake epicenter is not mentioned in the text, so I don't think it needs to be in this diagram.

Reply. We have included following description in the text:

"(see Fig. 1 for the epicenter of the 11-March-2011 earthquake and the FDNPS site)"

Ｌ150 The authors should describe the model domain in an easy-to-understand manner in terms of latitude and longitude.

Reply. We have included following description of the model domain:

“The model has a domain area from $125^{\circ}E$ to $155^{\circ}E$ in longitudes, and from $28^{\circ}N$ to $48^{\circ}N$ in latitudes.”

L171 The month of March 2011 is a period of large-scale atmospheric depression. Fig. 3(a) shows one month’s data at a time, but a more detailed analysis is needed.

Reply. We have included following analysis in the description:
“We note that the month of March 2011 is a period of large-scale atmospheric depression. Two large-scale low pressure systems are located on both side of the Bering Sea: The Aleutian Low on the left, and on the right is a surface low pressure system over the off-shore of area between the Bering Sea and the western Pacific coast of Canada.”

L213 The authors should include Fig. S2 in the text, as it is an important result. The results of the statistical analysis should also be described in more detail.

Reply. We have followed reviewer’s comment to include Fig. S2 in the main text, and include a description and a reference of the statistical method used in this work.

L245 Aoyama et al., 2016 is not in References; please cite 31 ad 40 more correctly. Also, there doesn’t seem to be any elevated Cs-137 measurements for this period in the cited sources.

Reply. We have corrected citation to Aoyama et al., 2016 (original cite 31). We have checked and corrected citations to 31 and 40. We have revised the description “The dose rates increased again from December 2011 to January 2012, during May-August and in October 2012; in January, March, and June-September 2013,…” (old, lines 241-242)

The Cs-137 measurements shown in Fig. 9 (and also in Fig. 2 of Aoyama et al. 2016 part 1 paper). There exists local maximum of Cs-137 of about 1.2E+04 Bq/m3 relative to nearby measurements of 0.8-0.9 Bq/m3 during December 2011 – January 2012. During May-August 2012, a local maximum of 0.9E+04 Bq/m3. In October 2012, a local maximum of 0.8E+04 Bq/m3. In January 2013, a local maximum of 1.6E+03 Bq/m3. In March 2013, a local maximum of 2.1E+03 Bq/m3. During June-September 2013, a local maximum of 5.5E+03 Bq/m3. During July-October 2014, a local

maximum of 3.1E+03 Bq/m3.

We have revised the following description:
“The dose rates increased again from December 2011 to January 2012, during May-August and in October 2012; in January, March, and June-September 2013; and in July-October 2014. Importantly, these are also periods showing existence of a local maximum of elevated ^{137}Cs measurements in the surface water of the FDNPS (see Fig.~\ref{fig10} and in Fig. 2 of Aoyama et al., 2016^{31}).”

L251 It is difficult for me to find out that the months of increase in the air dose rates mostly occur from November to April and from June to August in Fig 4 e. The authors should these periods more clearly.

Reply. We have included a schematic figure to highlight the months of increase in the air dose rates from time-series plot shown in Fig 4e.

L399 I think it is very unfortunate that the comparison is limited to Tokyo Bay only, despite the availability of data for the Pacific Ocean. For example, one of the authors has developed the North Pacific scale model presented in (Aoyama et al., 2016). I think a comparison with the North Pacific Scale Model would be very valuable.

Reply. We are very grateful to the reviewer for suggesting the use of the dose rate data over the North Pacific for verifying model results. The initial comparisons over the Tokyo Bay is encouraging. Previous modeling studies compared model results with the CTBTO data over the North Pacific. We are working on the follow-up work to compare model simulations with the air dose rates over the North Pacific. This would be very valuable, as commented by the reviewer.
Our measurements of the air dose rates over the land and over the water shows quite different characteristics. The mechanism of resuspension, as shown in Kajino et al. (2016), will be a key factor for assessing model capabilities in simulating the dispersions of the radionuclides after the FDNPS accident.

L503 In terms of resuspension, I think it is necessary to discuss whether it is the effect of the Fukushima accident or the effect from the continent due to atmospheric nuclear weapons tests.

Reply. We have added discussion of the effect from the continent due to the

atmospheric nuclear weapon tests. We have based our discussion on the radionuclide data collected and analyzed by the gamma spectrometers in the global radionuclide stations from the Comprehensive Nuclear-Test-Ban Treaty (CTBT) Organization (CTBTO) during the period 2000-2020. We have included the discussion using CTBTO data in section discussion.

Reviewer #2: The paper presents results of unique network of dose rate measurements done all over the world on board on merchant ships. However the interpretation, especially that regarding connection of North Atlantic results with post-accident trace releases from Fukushima seems to be for me not enough supported. The GM counter, as only dose rate meter is not sufficient tool for such long distance study.

Reply. We are very grateful to the reviewer for the comment.

The dose rate measurements over the northwestern Atlantic, as shown in Fig. 8(d) (revised manuscript) were faint, but were still positively correlated with the FDNPS emissions fluxes and the Tsukuba deposition fluxes. In the revised manuscript, we have used data from CTBTO for impacts of the FDNPS. The CTBTO measurements at Reykjavik provides good data from gamma spectrometer measurements of the CTBT-relevant radionuclides. We have included this description in the text.

[NOTE: If reviewer comments were submitted as an attachment file, they will be attached to this email and accessible via the submission site. Please log into your account, locate the manuscript record, and check for the action link "View Attachments". If this link does not appear, there are no attachment files.]

Reply. We have not found attachment file when logged in and checked for the action link “View Attachments”.

While revising your submission, please upload your figure files to the Preflight Analysis and Conversion Engine (PACE) digital diagnostic tool, https://pacev2.apexcovantage.com/. PACE helps ensure that figures meet PLOS requirements. To use PACE, you must first register as a user. Registration is free. Then, login and navigate to the UPLOAD tab, where you will find detailed instructions on how to use the tool. If you encounter any issues or have any questions when using

PACE, please email PLOS at figures@plos.org. Please note that Supporting Information files do not need this step.

Reply. We have uploaded all figures to PACE at https://pacev2.apexcovantage.com as suggested. The figure tiff files were checked and downloaded from PACE.

In compliance with data protection regulations, you may request that we remove your personal registration details at any time. (Remove my information/details). Please contact the publication office if you have any questions.

Reply. Thank you very much indeed.
