# Supplementary material for: Impacts on air dose rates after the Fukushima accident over the North Pacific from 19 March 2011 to 2 September 2015
Source: PLoS One. 2022 Aug 24;17(8):e0272937. doi: 10.1371/journal.pone.0272937 (PMC9401177; doi:10.1371/journal.pone.0272937)
Supplement: Attachment — Submitted filename: Response to Reviewers_2.pdf [file pone.0272937.s003.pdf]

Response to reviewers' comments. Manuscript Number PONE-D-21-14834R1

Impacts on air dose rates after the Fukushima accident over the North Pacific from 19 March 2011 to 2 September 2015

By Wang et al.

We are very grateful indeed to the reviewers, the editor, and the editorial office for very encouraging and insightful comments that have significantly increased the clarity of the manuscript. We have revised the manuscript following the comments. A detailed point to point response to each comment is given below.

Reviewer #2: Thank you for improving the text by introducing the changes suggested by reviewers. It a bit pity, that during improvements the numeration of rows disappeared from left side, and on right side it is cut out. This makes referring to certain places a bit difficult.

Reply. We have revised the line numbers to the left of the manuscript so that the row numbers can be viewed clearly.

Problematic sites remains:
In a section "The Monitoring Devices and Calibration" there are questions tags left in the brackets with references. This suggests that some reference is missing.

Reply. The questions tags were produced due to a missing numbers used in the cited reference which latex cannot find it. We have revised the cited reference numbers in the revised manuscript. (page 3, lines 75-76)

In the same section the measurement range is given only using maximum values (< 2mSv/h), whereas the minimum detectable value of 10 microSv/h is given a bit later. So I think the initially maximum value should be named for instance "the maximum detectable dose rate" or "the upper limit of measurement range"; not the "range" which apparently was from 10 micro to 2 mili Sv/h.

Reply. We have followed reviewer's comment to revise the description to "the upper limit of measurement range". (page 3, lines 74-76)

In a section "Emission and Deposition Fluxes" please note that "chi squared test" is

written with small character (“Chi” is not any name)

Reply. We have revised the Chi to chi, following reviewer’s comment. (page 6, line 191)
